# Emergency staged Whipple procedure for gastroduodenal mucormycosis: a rare life-saving intervention

**DOI:** 10.1093/jscr/rjag136

**Published:** 2026-03-11

**Authors:** Akash Raya, Rohit K Mishra, Alok Shrestha, Basant K Yadav, Baibhav Parajuli, Kritick Bhandari

**Affiliations:** Department of General Surgery, Chitwan Medical College, Bharatpur, Nepal; Department of General Surgery, Chitwan Medical College, Bharatpur, Nepal; Department of General Surgery, Chitwan Medical College, Bharatpur, Nepal; Department of General Surgery, Chitwan Medical College, Bharatpur, Nepal; Department of General Surgery, Chitwan Medical College, Bharatpur, Nepal; Department of Internal Medicine, KIST Medical College and Teaching Hospital, Gwarko, Lalitpur, Nepal

**Keywords:** Whipple’s procedure, gastrointestinal fungal infection, mucormycosis, gastroduodenal perforation

## Abstract

Gastrointestinal (GI) mucormycosis is a rare, angioinvasive disease with a high mortality rate. To prevent dissemination of infection, early surgical resections of affected areas and initiation of antifungals are necessary. Unfortunately, due to its rarity, diagnosis, and treatment are often delayed. Here, we have described a case of a 22-year-old with gastroduodenal mucormycosis. Initially radiological findings were thought to be a sequela of changes for gastric volvulus or pancreatitis. GI mucormucosis was later confirmed with tissue histopathology. He underwent staged emergency Whipple’s procedure and was started on antifungals. Fortunately, due to early intervention the patient recovered without complications.

## Introduction

Mucormycosis is a life threatening fungal infection that typically involves immunocompromised hosts and can affect any organ system. It is caused by fungi of the order Mucorales, previously known as Zygomycetes. Pulmonary, rhinocerebral, sinonasal, sino-orbital, and skin manifestations are the most common, accounting for >70% of cases. Gastrointestinal (GI) involvement is rare estimating 5%–13% of total mucormycosis cases [1]. We report this case to alert all the clinicians that gastrointestinal mucromycosis (GIM) can present with significant periampullary necrosis to an extent that a Whipple’s procedure may be needed.

## Case report

A 22-year-old man presented in our emergency room (ER) with pain in epigastric region for 2 months which was intermittent, dull aching and with no aggravating or relieving factors. Pain was described as being progressively worse and severe for 3 days. Other associated complains were melena for a month and shortness of breath for 3 days which gradually progressed to breathlessness at rest. There was no history of haematemesis, trauma, anorexia, and significant weight loss. He was diagnosed with pulmonary tuberculosis 1 month back at the time and was taking anti-tubercular medicine according to the national guidelines. He has a history of smoking (cigarette, marijuana) for 10 years accounting for 6 pack years and has been consuming alcohol every day for 10 years. His blood pressure was 60/40 mmHg and pulse rate was 140 beats per minute. Pallor and dehydration was evident. Abdomen was distended with generalized tenderness and guarding. Rebound tenderness was also elicited. On percussion, dull note was noted all over the abdomen. On auscultation bowel sounds were absent.

**Figure 1:**
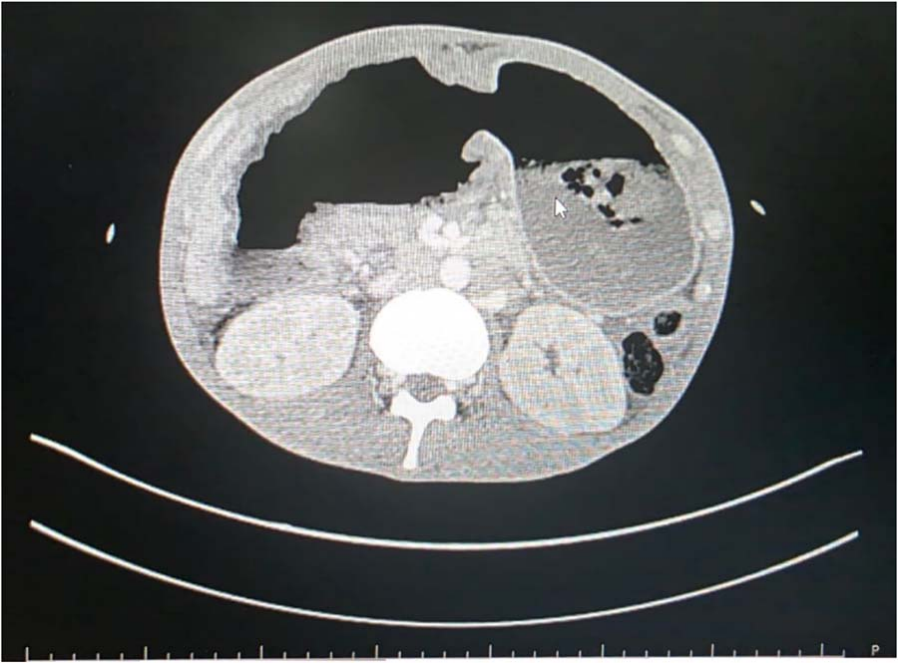
CECT scan showing non-enhancement of distal stomach.

Blood tests revealed low hemoglobin levels (2.1gm/dl), decreased white blood cells (WBC) count (38 840/mm^3^) as well as serum albumin levels (2.09 mg/dl). Contract enhanced computed tomography (CECT) scan revealed markedly distended stomach with hour-glass configuration, non-enhancement of the distal portion of the stomach (Fig. 1) and proximal (D1 and portion of D2) duodenum. Non-opacification of the contrast in gastroduodenal artery was noted. Obliteration of the distal most portion of the superior mesenteric vein (70%) and non-enhancement of the pancreatic neck was appreciated. Hypoperfusion of the pancreatic head was evident with moderate ascites. Initially findings were thought to be a sequela of changes for gastric volvulus or pancreatitis.

**Figure 2:**
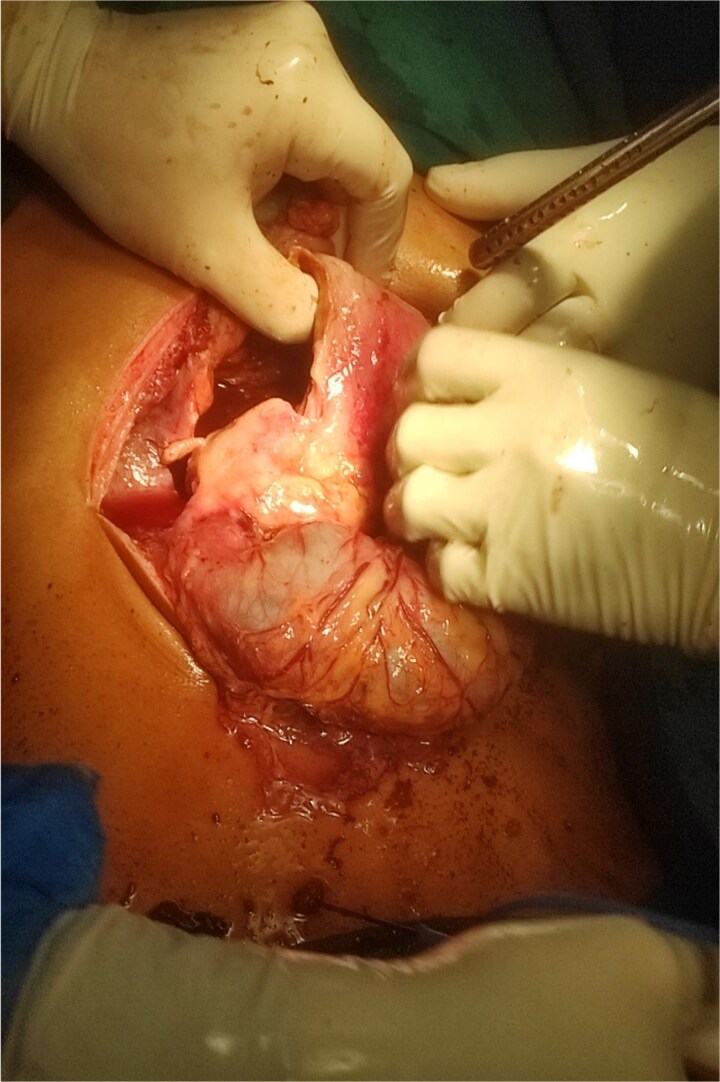
Intraoperative image showing gastroduodenal perforation and spillage of hemorrhagic content.

**Figure 3:**
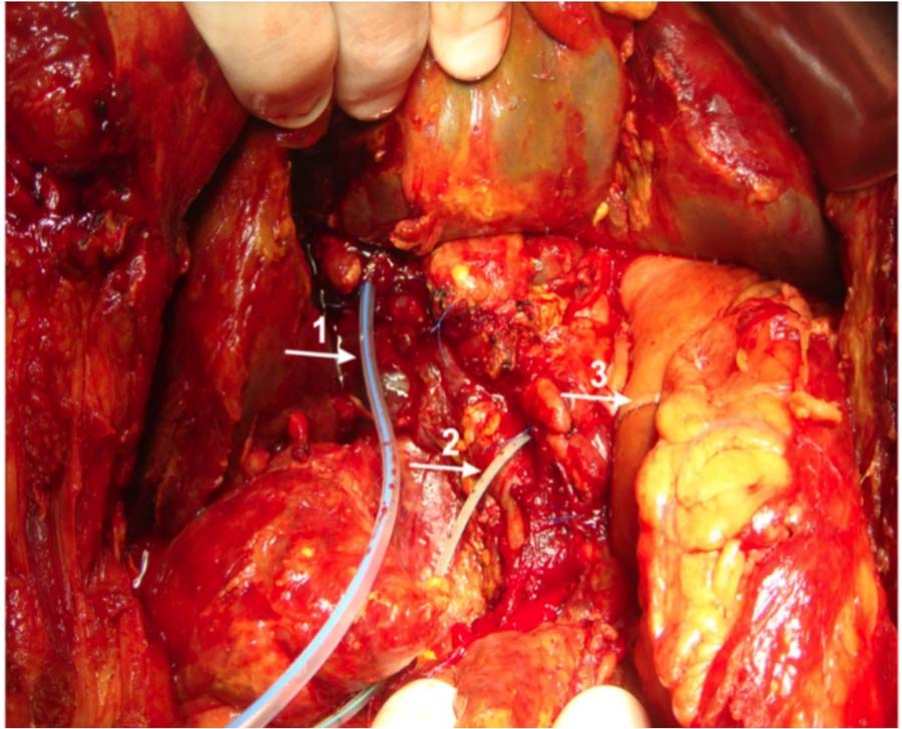
Intraoperative image showing placements of T-tube in common bile duct (1) and pancreatic drain in pancreatic duct (2). Gastrojejunal anastomosis is also visible (3).

**Figure 4:**
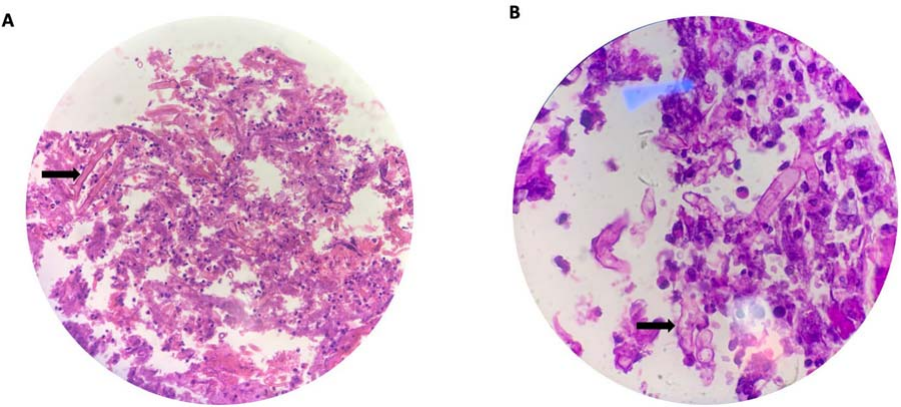
High power (40 X) view (A) and oil immersion high power (100 X) view (B) of broad ribbon like aseptate branching hyphae (arrows) seen with Periodic acid-Schiff (PAS) stain making an angle around 90° or greater consistent for microscopic features of mucormycosis.

Patient was a fluid non-responder and was kept on ionotropic support of noradrenaline at 0.33 micrograms/kg/min in the emergency room. Given the clinical presentation and hemodynamic instability we decided to plan for exploratory laparotomy and damage control surgery on Day 1. Intra operatively around 1 liter of foul smelling hemorrhagic content was suctioned. Perforation of size 7 × 10 cm at anterior wall of distal stomach and D1 with exposure of ampulla was visible (Fig. 2). Active bleeding from posterior wall of distal stomach was also evident. Anterolateral wall of stomach and D1 were densely adhered to inferior surface of liver and transverse colon. For damage control surgery, we proceeded with resection of gangrenous distal stomach, duodenum and segmental resection of transverse colon with exteriorization of proximal part with pancreaticobiliary drainage via ampulla with jejunostomy. However, given the extensive periampullary destruction, standard limited resections would not be enough and a staged Whipple’s procedure was necessary. Patient was shifted to the surgical intensive care unit under ventilator, noradrenaline, and cisatracurium support. On Day 2, noradrenaline was tapered down and discontinued, patient was on spontaneous continuous positive airway pressure mode with a Glasgow coma scale score of E_3_V_T_M_6_. Completion of staged Whipple’s with Roux-en-Y hepaticojejunostomy and gastrojejunostomy, and ileocolic anastomosis was carried out on Day 3 (Fig. 3). The patient was successfully extubated on post-operative day (POD) 1 of the second surgery under ventilator, noradrenaline and cisatrcurium support and moved to the inpatient surgical ward on POD 5 after being monitored in the intensive care unit (ICU). Specimen sent for histopathological examination disclosed fungal agents corresponding to mucormycosis (Fig. 4). Hence, a 10-day course of Amphotericin B was started. By POD 14, there were no complications, and subdiaphragmatic drains were removed. Patient presented on 25th POD after completion of his oral antifungals and the remaining right pelvic drain was also removed. On his latest visit, serum albumin levels were markedly low (1.9 g/dl) for which nutritional counselling was done. All other lab parameters such as total protein (6.5 g/dl), creatinine (0.4 mg/dl), urea (26 mg/dl), electrolytes, platelets (299 000/μl), and neutrophils (46.8%) were normal.

## Discussion

Mucormycosis is a life threatening fungal infection that typically involves immunocompromised hosts and can affect any organ system. GIM is rare (5%–13%) among cases reported for mucormycosis [1]. Common risk factors for gastrointestinal mucormycosis are diabetes, hemodialysis/peritoneal dialysis, malnutrition, alcohol use, corticosteroid therapy, and patients who have undergone liver/renal transplantation or surgery [2]. Although previously reported to affect immunocompromised, malnourished, or neonates there is a rising trend of cases of mucormycosis affecting immunocompetent adults as well [3, 5, 6]. The mode of transmission can be possibly ingestion of contaminated food in our case. Although the incidence of GIM is low, involvement of different parts of the GI tract has been reported as stomach being the most involved part (67%) followed by colon (21%). However small intestine (4%) and esophagus (2%) are less commonly reported [7].

GIM is a fulminant disease and progresses rapidly causing mortality in ~ 85% of the patient and only 25% of the cases are diagnosed ante-mortem [7]. The angioinvasive nature of GIM is closely tied to how the fungi grow. Mucor molds form hyphae in tissue. When spores start growing, these hyphae invade blood vessels, causing perforations and bleeding in gastrointestinal infections. Patients may present with bleeding, perforation, infarction, or peritonitis [1].

The choice of surgical treatment for gastric mucormycosis has traditionally been gastrectomy to decrease the chances of dissemination [5, 6]. In our case, a Whipple’s procedure was required because mucormycosis led to perforation of the periampullary duodenum. Mucormycosis causes bleeding leading to friable ischemic tissue that can’t be sutured together. As the infection also spread to D2, pancreaticoduodenectomy was the only way to fully remove the infection and the devitalized tissue without the risk of future leaks. Only Vadi et al. describes a case of GIM that underwent Whipple’s procedure without mortality. As a result of dissemination, a right total nephrectomy was performed as well. It is remarkable that a 65 years old patient was able to survive such an extensive procedure given her history of recent COVID infection and hypoalbuminemia preoperatively. Liposomal amphotericin B (LamB) is the drug of choice for the treatment of mucormycosis [8]. Mortality rates for LamB monotherapy is 50% to 61% and combining LamB with surgery lowers this to 19% to 44% [4]. Combination of timely diagnosis and surgical intervention with antifungal drugs is the key to produce optimal patient outcomes.

## Data Availability

Data sharing is not applicable to this article.

## References

[1] Henry B, Lefevre Utile A, Jaureguiberry S et al. Gastrointestinal and intra-abdominal mucormycosis in non-haematological patients—a comprehensive review. J Fungi 2025;11:298. 10.3390/jof11040298 PMC12028458 40278118

[2] Addasi Y, Nguyen AH, Sabri A et al. Gastrointestinal mucormycosis: a clinical review. Gastroenterology Res 2023;16:249–53. 10.14740/gr1662 37937225 PMC10627358

[3] Huang H, Xie L, Zheng Z et al. Mucormycosis-induced upper gastrointestinal ulcer perforation in immunocompetent patients: a report of two cases. BMC Gastroenterol 2021;21:311. 34404350 10.1186/s12876-021-01881-8 PMC8370051

[4] Prakash H, Chakrabarti A. Epidemiology of mucormycosis in India. Microorganisms. 2021;9:523. 33806386 10.3390/microorganisms9030523 PMC8000977

[5] Termos S, Othman F, Alali M et al. Total gastric necrosis due to mucormycosis: a rare case of gastric perforation. Am J Case Rep 2018;19:527–33. 10.12659/AJCR.908952 29724988 PMC5956728

[6] de ABFBB, Duarte ML, Dos SLR et al. A rare case of gastric mucormycosis in an immunocompetent patient. Rev Soc Bras Med Trop 2018;51:401–2. 29972579 10.1590/0037-8682-0304-2017

[7] Ralaizanaka BM, Razafindrazoto CI, Bolot E et al. Gastrointestinal mucormycosis-induced massive lower gastrointestinal bleeding, rectal perforation, and pulmonary embolism: a long diagnostic pathway in a case report. Clin Exp Gastroenterol 2022;15:145–51. 10.2147/CEG.S373728 35983373 PMC9381012

[8] Vadi S, Raut A, Shah S et al. Post coronavirus disease- 19 invasive renal and gastrointestinal mucormycosis. Indian J Med Microbiol 2022;40:462–4. 10.1016/j.ijmmb.2022.03.006 35527119 PMC9072856

